# The Sirt3–CD38 axis induces mitochondrial dysfunction in hypertrophied heart by regulating mitochondrial calcium overload

**DOI:** 10.1186/s40001-025-03211-0

**Published:** 2025-10-14

**Authors:** Jia Liu, Ning Liu, Chao Qi, Delu Dong, Bin Liu

**Affiliations:** 1 Department of Cardiology, The Second Hospital of Jilin University, Changchun, 130041 China; 2 Laboratory Teaching Center of Basic Medicine, Jilin University, Changchun, 130021 China

**Keywords:** Cardiac hypertrophy_1_, Sirt3_2_, ROS_3_, CD38_4_, Mitochondria_5_

## Abstract

Mitochondrial dysfunction driven by calcium overload is a hallmark of cardiac hypertrophy, yet the role of Sirtuin-3 (Sirt3) in regulating this process remains incompletely defined. Specifically, the mechanism by which CD38-mediated NAD depletion links Sirt3 deficiency to mitochondrial calcium dysregulation remains incompletely elucidated. Therefore, 12 week-old Sirt3-deficient mice were used as cardiac hypertrophy model. The morphological changes of cardiac muscle fibers and the ultra-structure changes of mitochondria were detected by hematoxylin and eosin (HE) staining and transmission electron microscopy (TEM). Then, multi-omics approach was used to analyze the differently expressed genes and different metabolites. Key genes and metabolites were scrutinized through Gene Ontology (GO), Kyoto Encyclopedia of Genes and Genomes (KEGG). Finally, in vitro studies examining the effects of Sirt3 knockdown on H9C2 cells, including intracelluler and mitochondrial reactive oxygen species (ROS) and calcium, and mitochondrial membrane potential (MMP). Western blot and qPCR were used to verify the differently expressed genes. The hearts of Sirt3-deficient mice increased myofiber thickness, and altered mitochondrial morphology. Sirt3 deficiency induced mitochondrial dysfunction was promoted by an inhibition of the translation of oxidative phosphorylation (OXPHOS) complex subunits. Multi-omics profiling implicated CD38 as a major NAD consumer and linked the metabolites of CD38 to cAMP signaling pathways. Furthermore, in vitro studies examining H9C2 Sirt3 knockdown showed an increase in intracellular and mitochondrial ROS levels, a decrease in MMP, and promoted MCU expression and mitochondrial calcium overload. However, CD38 inhibitors effectively attenuated Sirt3 knockdown-induced elevations in intracellular and mitochondrial ROS levels, dissipation of mitochondrial membrane potential, and mitochondrial calcium overload, thereby restoring mitochondrial function. In summary, the Sirt3–CD38 axis induces mitochondrial dysfunction in hypertrophied heart by regulating mitochondrial calcium overload. These findings will aid in providing new ideas for the prevention and treatment of age-related cardiac hypertrophy.

## Introduction

Cardiac hypertrophy is a complex multifactorial syndrome, which makes it difficult to determine effective therapeutic targets [1]. Cardiac hypertrophy involves complex interplay between oxidative stress and dysregulated mitochondrial Ca^2^⁺ metabolism [2–4]. In angiotensin II induced hypertrophy model, mitochondrial Ca^2^⁺ overload was driven by upregulated mitochondrial calcium uniporter (MCU), which induced electron transport chain (ETC) dysfunction and excessive ROS production [5–7]. Critically, silencing MCU reverses mitochondrial dysfunction by normalizing Ca^2^⁺ flux and suppressing ROS, highlighting MCU as a pivotal node linking both pathways [6–8]. Therefore, further exploration of key molecules that affect the relationship between these two factors may provide new therapeutic targets for the treatment of myocardial hypertrophy.

Sirt3 is an NAD-dependent deacetylase located in mitochondria, which plays a critical role in regulating mitochondrial redox homeostasis, Ca^2^⁺ homeostasis, energy metabolism, protein input, and other processes [9–12]. Mitochondrial Sirt3 and its cofactor, NAD, steadily decrease with age, and are of interest in age-related cardiomyocyte hypertrophy [13–18]. This indicates that Sirt3 plays an indispensable role in the process of myocardial hypertrophy. Therefore, mitochondrial dysfunction caused by Sirt3 depletion and deficiency leads to the development of various cardiovascular diseases [19]. However, the mechanism by which Sirt3 regulates the interaction between oxidative stress and mitochondrial Ca^2^⁺ overload during myocardial hypertrophy is incompletely defined.

In recent years, it has been shown that Sirt3 can interact with other NAD-consuming enzymes, such as CD38 and ADP ribose oligomerases (PARPs), to affect the process of myocardial hypertrophy [20–23]. In addition, CD38 and Sirt3 consumes NAD and produces nicotinamide and O-acetyl-ADP-ribose (OAADPr). Nicotinamide is an endogenous sirtuins inhibitor, and OAADPr may regulate gene silencing by facilitating the assembly and loading of the Sir2–4 silencing complex onto nucleosomes [24, 25]. Therefore, the expression of other NAD-consuming enzymes may be regulated by Sirt3 metabolites. It has been shown that CD38 degrades NAD to produce cyclic ADP-ribose (cADPR), which is an important molecule for intracellular Ca^2+^ mobilization for regulating the cAMP signaling pathway [20, 23]. This suggests that NAD-consuming enzymes such as Sirt3 and CD38 may regulate oxidative stress and mitochondrial Ca^2+^ levels through their metabolites. Therefore, in-depth exploration of the interaction mechanism between Sirt3 and CD38 will help elucidate the interdependent mechanisms of oxidative stress and abnormal mitochondrial Ca^2+^ overload in myocardial hypertrophy.

## Materials and methods

### Animal models

Twelve week-old wild-type male C57BL/6 J mice were purchased from Beijing Vital River Laboratory Animal Technology Co., Ltd. (Beijing, China), and Sirt3-deficient (Sirt3 ko) mice (#027975, B6.129S6(Cg)–Sirt3tm 1.1 F w a/J) were purchased from the Jackson Laboratory (Bar Harbor, ME, USA). Mice were breed in barrier facilities at the College of Basic Medical Sciences (Jilin University, Changchun, China). All animals were housed in a pathogen-free environment under controlled conditions (12 h light–dark cycles) and given food and water adlibitum. All animal procedures were approved by the Animal Experimentation Ethics Committee of the College of Basic Medical Sciences (Jilin University).

### Cell culture and transfection

H9C2 cells were acquired from the National Collection of Authenticated Cell Cultures (Shanghai, China) and cultured in DMEM (Gibco, Carlsbad, CA, USA) supplemented with 10% fetal bovine serum (Clark Bioscience, Virginia, USA), 100 U/mL penicillin, and 100 mg/mL streptomycin (Beyotime Biotechnology, Shanghai, China). Cells were maintained at 37 °C, 95% humidity, and 5% CO_2_ in an incubator (Thermo Fisher Scientific, Waltham, Massachusetts, USA). H9C2 cells were transfected with Sirt3 siRNA (Forward: 5′–3′: CUUGUCUGAAUCGGUACAGAATT, Reverse: 5′–3′: UUCUGUACCGAUUCAGACAGTT) for 24 h to knockdown its expression.

### Measurement of intracellular and mitochondrial reactive oxygen species (ROS)

The CD38 inhibitor (78C, 5 nM) treated H9C2 (si-Sirt3) cells were rinsed with PBS and incubated with 1 ml DCFH–DA (10 μM; Beyotime Biotechnology, Shanghai, China) or 1 ml MitoSox green (1 μM; Thermo Fisher Scientific, MA, USA) for 20 min at 37 °C in the dark. After DCFH–DA and MitoSox green staining, the cells were rinsed 3 times with PBS, detached with 0.25% trypsin without EDTA, evaluated using an EasyCyte flow cytometer and analyzed using Guava 3.1.1 software (Merck Millipore, MA, USA).

### Mitochondrial membrane potential (MMP)

H9C2 (si-Sirt3) cells treated with The CD38 inhibitor (78C, 5 nM) were rinsed with PBS, and incubated with 1 ml JC-1 (Beyotime Biotechnology, Shanghai, China) working solution according to the manufacturer’s protocols. Following incubation, cells were rinsed 2 times with dye buffer, detached using 0.25% trypsin without EDTA, analyzed using EasyCyte flow cytometer and quantified using Guava 3.1.1 software (Merck Millipore, MA, USA).

### ***Measurement of intracellular Ca***^***2***+^***and mitochondrial Ca***^***2***+^

Fluo-4 and Rhod2 (Yeasen Biotechnology, Shanghai, China) were separately used to measure intracellular Ca^2+^ and mitochondrial Ca^2+^. Cells were incubated with 1 µM Fluo-4 or Rhod2 for 30 min at 37 °C, and then washed twice with serum-free medium before detection. Then, the cells were digested by 0.25% trypsin without EDTA (Servicebio Technology, Wuhan, China) and collected cells, and washed with PBS once. Finally, EasyCyte flow cytometer was used to detect the Fluo-4 and Rhod2 fluorescence intensity, which was quantified using Guava 3.1.1 software (Merck Millipore, MA, USA).

### ***Quantitative real-time PCR ***[27]

Total RNA from heart tissue was extracted using Trizol reagent (Invitrogen). cDNA was synthesized from the RNA products using the Omniscript Reverse Transcription Kit (QIAGEN). RT-qPCR was performed to estimate mRNA levels using SYBR SuperMix (TransGen Biotech, Beijing, China). All reactions were performed twice using a Bio-Rad CFX96 Touch Real-Time PCR System (Bio-Rad). Glyceraldehyde-3-phosphate dehydrogenase (GAPDH) was used as an internal control. The relative expression level for each gene was calculated using the 2^−ΔΔCt^ relative quantification method and normalized to the endogenous level. The primer sequences used are listed in Table 1.

**Table 1:** Primer sequences used for qPCR (Primers 5′–3′)

Gene	Forward	Reverse
Gapdh	GTCGTGGAGTCTACTGGTGTC	GAGCCCTTCCACAATGCCAAA
mt-Nd1	TCTGCCAGCCTGACCCATAG	CCGGCTGCGTATTCTACGTT
mt-Co2	GCCGACTAAATCAAGCAACA	CAATGGGCATAAAGCTATGG
mt-Atp6	AATTACAGGCTTCCGACACAAAC	TGGAATTAGTGAAATTGGAGTTCCT
Ndufv1	GTGGCTCATCTACTCCACTGA	TCGAGCGATCCATAACAATAA
Sdhc	GCTGCGTTCTTGCTGAGACA	ATCTCCTCCTTAGCTGTGGTT
Uqcrc2	AAAGTTGCCCCGAAGGTTAAA	GAGCATAGTTTTCCAGAGAAGCA

### Protein quantification and western blot

WT mice (*n* = 3) and Sirt3 ko mice (*n* = 3) heart tissue and H9C2 cells were lysed in RIPA lysis buffer (Beyotime Biotechnology) containing phenylmethanesulfonylfluoride (PMSF, Invitrogen, Waltham, MA, USA). Following ultrasonication, samples were centrifuged for 10 min at 10000 × *g* and 4 °C. The supernatants were transfer to a new 1.5 ml tube and quantified using a BCA protein assay kit (Beyotime Biotechnology) according to the manufacturer’s protocols. Next, 5 µg of each protein sample were separated via SDS–PAGE and then transferred to a PVDF membrane. The membrane was blocked with 5% skim milk powder in TBST for 2 h at room temperature. Samples were then incubated with primary antibodies, including anti-beta actin monoclonal antibody (66009-1-Ig), anti-SIRT3 polyclonal antibody (10099-1-AP), anti-ATP synthase 8 (MT-ATP8) polyclonal antibody (26723-1-AP), and anti-CD38 monoclonal antibody (60006-1-Ig, Proteintech Group), and anti-mitochondrially encoded cytochrome C oxidase I (MT-CO1) mouse monoclonal antibody (PTM 5109), anti-ATP synthase F1 subunit alpha (ATP5A1) mouse monoclonal antibody (PTM 5163, Jingjie PTM Biolab) 4 ℃ overnight, samples were incubated with corresponding HRP-labeled secondary antibodies for 1 h at room temperature. Immunodetection was carried out using ECL reagent according to the manufacturer and imaged using a Syngene Bioimaging system (Synoptics, Ltd., Cambridge, UK).

### ***Histological and morphological quantitative analysis of muscle fibers ***[27]

The WT mice (*n* = 3) and Sirt3 ko mice (*n* = 3) hearts were fixed with 4% paraformaldehyde, embedded in paraffin, dehydrated, and rehydrated before staining coronal and sagittal sections with hematoxylin and eosin (HE). Finally, we observed the morphological characteristics of the muscle fibers using a microscope (CKX53; Olympus, Japan). Quantitative morphological analysis of the myocardial fibers was performed using ImageJ software (Version 1.52a; http://rsb.info.nih.gov/ij/, accessed on 3rd August 2022) using images of the HE-stained cross sections.

### Mitochondrial morphology

To detect the effect of Sirt3 on mitochondrial morphology, WT mice (*n* = 3) and Sirt3 ko mice (*n* = 3) heart samples were sectioned (1 mm^3^), fixed with 2.5% glutaraldehyde, and post-fixed with 1% osmium tetroxide (OsO_4_) in 0.1 M phosphate buffer (PB, pH 7.4) for 2 h at room temperature in the dark. Samples were rinsed 3 times in 0.1 M PB (pH 7.4) and dehydrated at room temperature with an ethanol gradient (30%, 50%, 70%, 80%, 95%, 100%, 100%; 20 min each), followed by two changes in acetone for 15 min. Samples were then embedded in an acetone/epoxy resin (EMBed 812) mixture at 37 °C with the following ratios: a 1:1 for 2–4 h; a 1:2 overnight; and pure resin for 5–8 h. The samples were then transferred to embedding models with fresh pure resin and stored at 37 °C overnight. Polymerization was then performed at 65 °C for more than 48 h. The resin blocks were then sectioned (60–80 nm) on an ultramicrotome and the tissues were fished onto 150 meshes cuprum grids with formvar film. The mesh was then stained with 2% uranium acetate saturated alcohol solution for 8 min in the dark, rinsed 3 times in 70% ethanol and then rinsed 3 times in ultrapure water. The samples were then incubated with 2.6% lead citrate for 8 min to avoid CO_2_ staining, and then rinsed 3 times with ultrapure water. Drying was completed using filer paper, and the cuprum grids were put into the grid board and dried overnight at room temperature. The sections were then observed using transmission electron microscopy (TEM).

### RNA-sequencing (RNA-seq) analysis

Total RNA was isolated from the WT mice (*n* = 3) and Sirt3 ko mice (*n* = 3) heart samples using Trizol reagent (Invitrogen, MA, USA). Samples were then treated with DNase and RNA quantification and purity NanoDrop spectrophotometer (Thermo Scientific, MA, USA). A total of 3 μg RNA per sample was used as input material for the RNA sample preparations. Briefly, mRNA was purified from total RNA using poly-T oligo-attached magnetic beads.

Fragmentation was completed using divalent cations under an elevated temperature in Illumina fragmentation buffer (NEB, Ipswich, MA, USA). First strand cDNA was synthesized using random oligonucleotides, while second strand cDNA was synthesized using DNA Polymerase I and RNase H. Any remaining overhangs were blunted using exonuclease/polymerase. To select preferred cDNA fragments (400–500 bp), the library fragments were purified using the AMPure XP system (Beckman Coulter, Beverly, CA, USA). The library was then sequenced on an Illumina NovaSeq 6000 platform (NEB) by Shanghai Personal Biotechnology Co. Ltd and differential expression was determined using DESeq2 (v.1.30.3) with Bonferroni correction. Genes with p < 0.05 and |fold change (FC)|> 1.5 were deemed differentially expressed.

### 4D label-free proteomic analysis

Proteins were extracted from WT mice (*n* = 3) and Sirt3 ko mice (*n* = 3) heart samples using SDT (4% SDS, 100 mM Tris–HCl, 1 mM DTT, pH 7.6). Samples were then trypsin digested and desalted on C18 Cartridges [Empore^™^ SPE Cartridges C18 (standard density), bed I.D. 7 mm, volume 3 ml, Sigma], concentrated by vacuum centrifugation and reconstituted in 40 µl of 0.1% (v/v) formic acid. After detergent treatment, peptide content was estimated based on UV light spectral density at 280 nm, with an extinctions coefficient of 1.1 of 0.1% (g/l) solution calculated on the basis of tryptophan and tyrosine frequencies in vertebrate proteins.

Liquid chromatography–tandem mass spectrometry (LC–MS/MS) was performed on a Nanoelute nanoflow LC system (Bruker Daltonics, Bremen, Germany) coupled to a trapped ion mobility–quadrupole time-of-flight mass spectrometer (timsTOF Pro mass spectrometer; Bruker Daltonics) for 60/120/240 min. The peptides were loaded onto a reverse phase trap column (Acclaim PepMap100, 100 μm × 2 cm, nanoViper, C18; Thermo Scientific, Waltham, MA, USA) connected to a C18 reverse-phase analytical column (Easy Column, 10 cm long, 75 μm inner diameter, 3 μm resin; Thermo Scientific) in buffer A (0.1% Formic acid) and separated with a linear gradient of buffer B (84% acetonitrile and 0.1% Formic acid) at a flow rate of 300 nl/min controlled by IntelliFlow technology. The mass spectrometer was operated in positive ion mode and collected ion mobility MS spectra over a mass range of m/z 100–1700 and ion mobility range (1/K0) of 0.6–1.6. Ten cycles of parallel accumulation serial fragmentation (PASEF) MS/MS with a target intensity of 1.5k and a threshold of 2500 were performed. An active exclusion release time of 0.4 min was also enabled. Finally, the raw MS data were evaluated using MaxQuant (v.1.5.3.17).

### Metabolomics analysis

Frozen WT mice (*n* = 3) and Sirt3 ko mice (*n* = 3) heart samples (100 mg) were homogenized with 200 μL of ddH_2_O and five ceramic beads using a homogenizer. Metabolites were then extracted by adding 800 μL methanol/acetonitrile (1:1, v/v), centrifuging (14000 × *g*, 4 °C, 20 min), and drying the supernatant via vacuum centrifugation. For LC–MS analysis, the samples were re-dissolved in 100 μL acetonitrile/water (1:1, v/v) solvent and centrifuged at 14000 × *g* at 4 °C for 15 min. Analysis was performed by Shanghai Applied Protein Technology Co., Ltd. using ultra-high-performance liquid chromatography (UHPLC; 1290 Infinity LC, Agilent Technologies, Santa Clara, CA, USA) coupled with quadrupole time-of-flight MS (Q-TOF–MS; TripleTOF 6600, AB Sciex, Framingham, MA, USA). Hydrophilic interaction chromatographic (HILIC) separation was carried out on an Acquity UPLC BEH Amide column (2.1 mm × 100 mm, 1.7 µm, Waters, Ireland). Electrospray ionization (ESI) was conducted in both positive and negative acquisition modes, with mobile phase A containing 25 mM ammonium acetate and 25 mM ammonium hydroxide in water and mobile phase B containing acetonitrile.

After sum-normalization, the processed data were analyzed using an R package (ropls) for multivariate data analysis that included Pareto-scaled principal component analysis (PCA) and orthogonal partial least-squares discriminant analysis (OPLS–DA). Model robustness was determined using sevenfold cross-validation and response permutation testing. The variable importance in the projection (VIP) value was calculated for each variable in the OPLS–DA model to determine its contribution to the classification. A Student’s *t* test was applied to identify significant differences between two groups of independent samples, with metabolites deemed significant at VIP > 1 and *p* < 0.05.

### Bioinformatics analysis

Hierarchical clustering of differentially expressed genes, proteins, and metabolites was performed using Heatmapper. Gene Ontology (GO) and Kyoto Encyclopedia of Genes and Genomes (KEGG) enrichment analyses were also performed to determine the main biological functions and pathway associations for the identified DEGs and DEPs. To analyze overlapping DEGs and differentially expressed proteins (DEPs), a Venn diagram was constructed (https://bioinfogp.cnb.csic.es/tools/venny/index.html). To explore potential protein–protein interactions (PPIs) associated with identified DEPs, the data were imported into the EMBL Search Tool for the Retrieval of Interacting Proteins (STRING) database v 12.0 (http://string.embl.de/) and an interaction map was generated.

### Statistical analysis

All results are given as mean values ± standard deviation (SD), with means based on data from at least three independent experiments. Statistical differences between mean values for each group were determined using a unpaired two-tailed Student’s *t* tests in GraphPad Prism 8.0.2 (GraphPad Software, CA, USA), with *p* < 0.05 considered statistically significant.

## Results

### Sirt3 deficiency induces cardiac hypertrophy

**Fig. 1:**
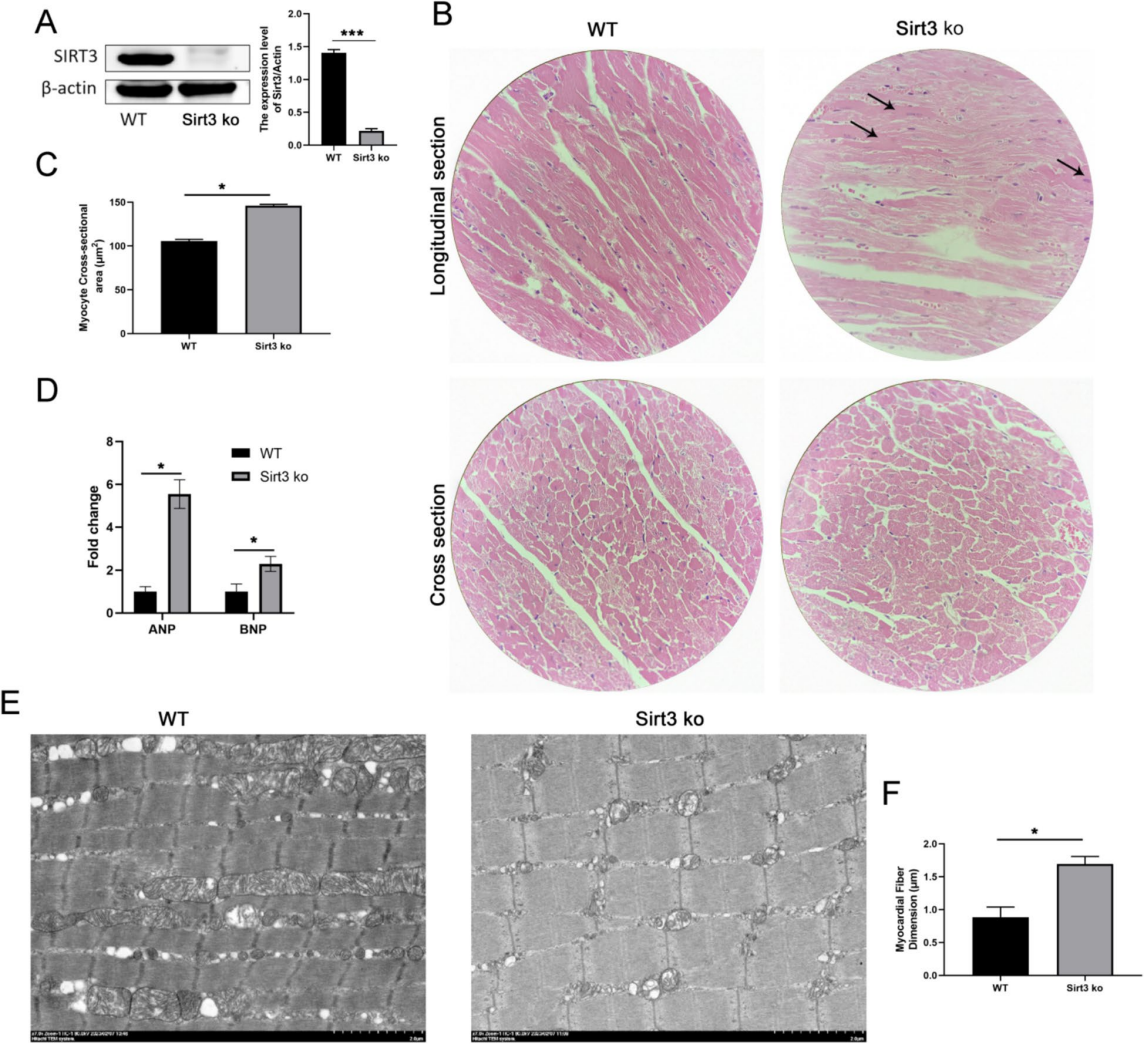
Sirt3 deficiency promotes cardiac hypertrophy. **A** Sirt3 protein expression in the myocardium of wild-type and Sirt3-deficienct (Sirt3 ko) mice as detected by Western blot. **B** HE staining examining myofiber thickening to evaluate cardiac hypertrophy (400 ×). **C** Statistical data of myocyte cross-sectional area. **D** Cardiac hypertrophy markers ANP and BNP were detected by transcriptomics (RNA-seq). **E** Myocardial fiber morphology detected by transmission electron microscopy (TEM). **F** Myocardial fiber diameter of WT and Sirt3 ko mice was measured in ImageJ software. Statistical significance: *p < 0.05

Previous studies have shown that the expression of Sirt3 is downregulated in hypertrophic cardiac tissue [15, 21]. To confirm the relationship between Sirt3 decline and cardiac hypertrophy, Sirt3-deficiency (Sirt3-ko) mice were used as the cardiac hypertrophy model. First, western blot was used to detect the knockout efficiency, and the results showed a significant decrease in Sirt3 expression in the Sirt3-ko mice heart tissue relative to the wild-type controls (Fig. 1A). Second, the myocyte cross-sectional area increased significantly in the Sirt3-ko mice as shown in HE staining (Fig. 1B, C). Third, the RNA-seq results showed that cardiac hypertrophic marker genes, ANP and BNP, were significantly upregulated in the Sirt3-ko mice (Fig. 1D). Finally, the results of TEM showed that the diameter of myocardial fibers was significantly increased in the Sirt3-ko mice heart compared with wide type mice (Fig. 1E, F). All of these results indicate that Sirt3 deficiency may induce cardiac hypertrophy.

### *Sirt3 deficiency promotes cardiac hypertrophy *via* impairing mitochondrial function*

**Fig. 2:**
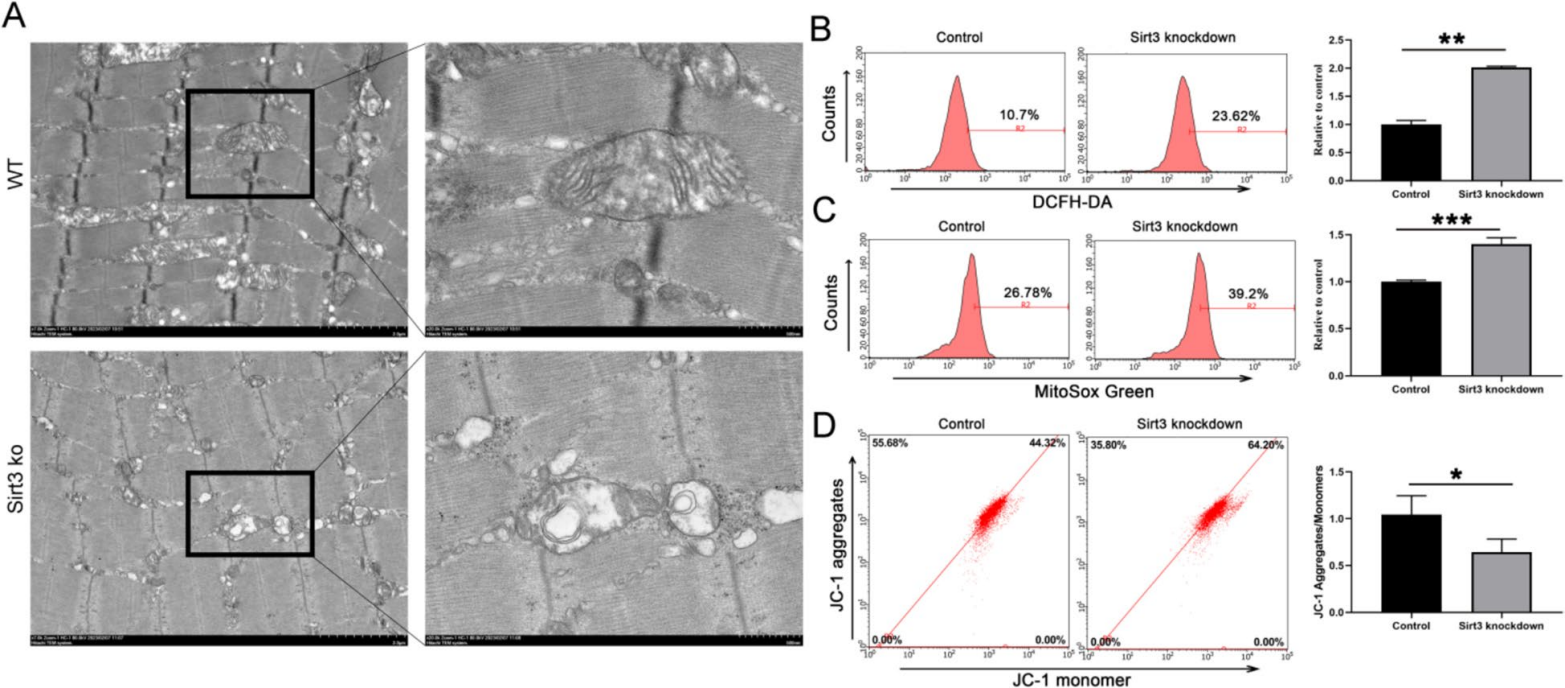
Sirt3 deficiency promotes cardiac hypertrophy via oxidative stress induced by mitochondrial dysfunction. **A** Mitochondrial morphology detected by transmission electron microscopy (TEM). H9C2 cells were transfected with Sirt3 siRNA for 24 h, and then, H9C2 cells were labeled with (**B**) DCFH–DA and (**C**) MitoSox green to determine total ROS levels and mitochondrial ROS levels, or (**D**) JC-1 to assess the MMP via flow cytometry. Statistical significance: *p < 0.05, **p < 0.01, ***p < 0.001

To further confirm that Sirt3 deficiency could induce mitochondrial dysfunction, TEM was also used to characterize mitochondrial morphological changes in Sirt3-deficient myocardial cells. In the wild-type heart tissue, the mitochondria were abundant with an elongated or round morphology and contained extensive inner mitochondrial membranes with abundant cristae (Fig. 2A). In contrast, Sirt3-deficient mice displayed predominantly absent cristae, and possessed a vacuole-like mitochondrial matrix (Fig. 2A). To further explore the effects of Sirt3 on mitochondrial function, H9C2 Sirt3 knockdown cells were constructed and the level of intracellular and mitochondrial ROS and mitochondrial membrane potential was examined using flow cytometry. The results showed that Sirt3 knockdown significantly increases the level of intracellular and mitochondrial ROS (Fig. 2B, C), and significantly decreases the mitochondrial membrane potential (Fig. 2D). All of these results indicate that Sirt3 deficiency promotes cardiac hypertrophy by impairing mitochondrial function.

### *Sirt3 deficiency induces mitochondrial dysfunction *via* inhibiting the translation of oxidative phosphorylation complex subunits*

**Fig. 3:**
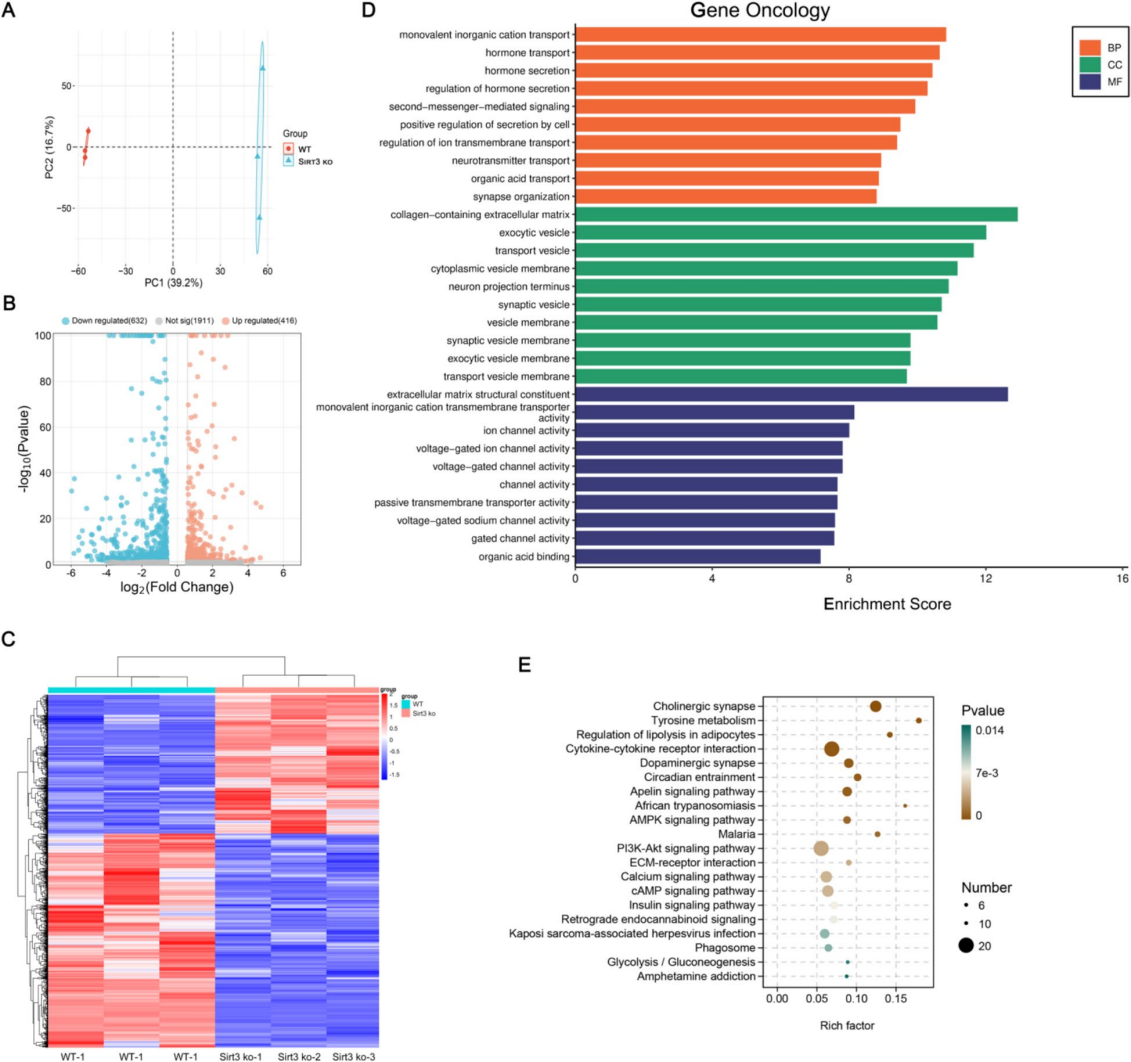
Transcriptional profiles for Sirt3-deficienct and wild-type mice hearts following RNA-seq analysis. **A** Principal component analysis (PCA) examining Sirt3-deficient and wild-type samples. **B** Volcano plot highlighting the DEGs between the two groups. **C** Heatmap showing the expressional profiles of each group. **D** GO enrichment of DEGs within 3 functional groups: biological processes (BP), cellular components (CC) or molecular functions (MF). **E** KEGG pathway analysis was used to reveal the pathways related to DEGs

To investigate the effect of Sirt3 on global cardiomyocyte transcription and translation, a multi-omics approach was utilized. First, mRNA expression in Sirt3-deficient and wild-type mice hearts were examined using RNA-seq. Following PCA analysis, the two groups were well-separated (Fig. 3A). A total of 1048 deferentially expressed genes (DEGs), including 416 upregulated DEGs and 632 downregulated DEGs, were identified (p < 0.05, log2FC ≥ 1.5; Fig. 3B) and a heatmap was constructed to visualize expression profiles (Fig. 3C). The identified DEGs were then examined using Gene Oncology (GO) analysis (Fig. 3D) and the highest enrichment levels were noted in monovalent inorganic cation transport (biological process), ion channel activity (cell component), and voltage-gated ion channel (molecular function). Furthermore, Kyoto Encyclopedia of Genes and Genomes (KEGG) pathway enrichment analysis showed that these DEGs were predominantly enriched in AMPK, PI3K–Akt, calcium, and cAMP signaling pathways (Fig. 3E).

**Fig. 4:**
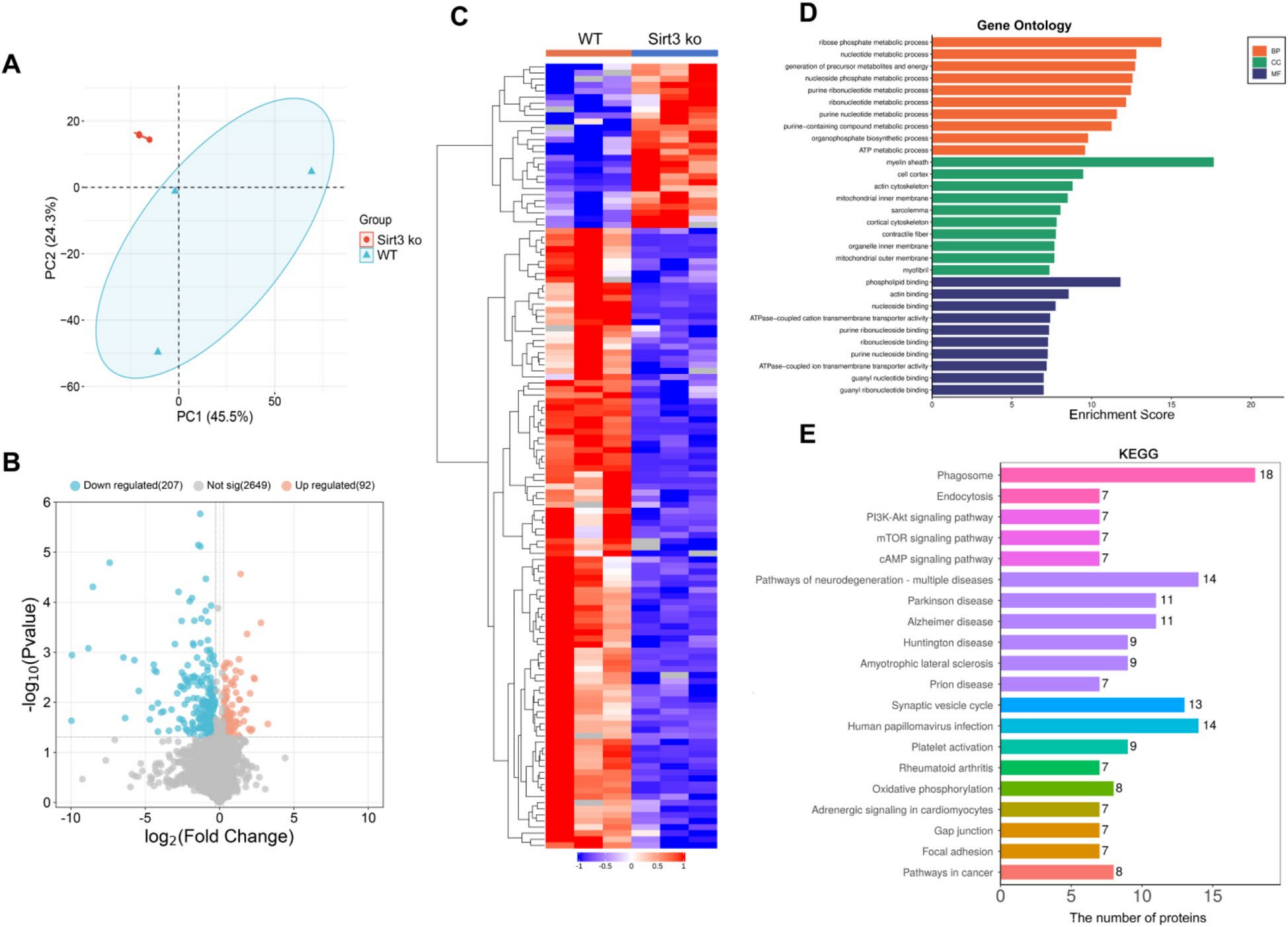
Proteomic profiles for Sirt3-deficienct and wild-type mice hearts following LC–MS/MS analysis. **A** Principal component analysis (PCA) of Sirt3-deficient and wild-type samples. **B** Volcano plot highlighting the DEPs between the two groups. **C** Heatmap showing the expressional profiles for each group. **D** GO enrichment of DEPs within 3 functional groups: biological processes (BP), cellular components (CC) or molecular functions (MF). **E** KEGG pathway enrichment with the number of DEPs per term indicated

To evaluate the myocardial proteomic profiles for Sirt3-deficient and wild-type mice, LC–MS/MS was employed. PCA results showed two distinct groups as it did for the RNA-seq data (Fig. 4A). A total of 299 deferentially expressed proteins (DEPs), 92 upregulated and 207 downregulated, were identified (Fig. 4B) and further visualized by constructing a heatmap (Fig. 4C). GO analysis showed that the DEPs were associated with ATP metabolism (biological process), mitochondrial inner and outer membrane (cell component), and ATPase-coupled ion transmembrane transport activity (molecular function) (Fig. 4D). Of the enriched KEGG pathway, the pathways of interest included PI3K–Akt signaling, oxidative phosphorylation, cAMP signaling, and mTOR signaling (Fig. 4E). Both the transcriptomic and proteomic results implicate mitochondrial energy metabolism and ion transport as factors that may play a vital role in cardiac hypertrophy that is associated with a Sirt3 deficiency.

**Fig. 5:**
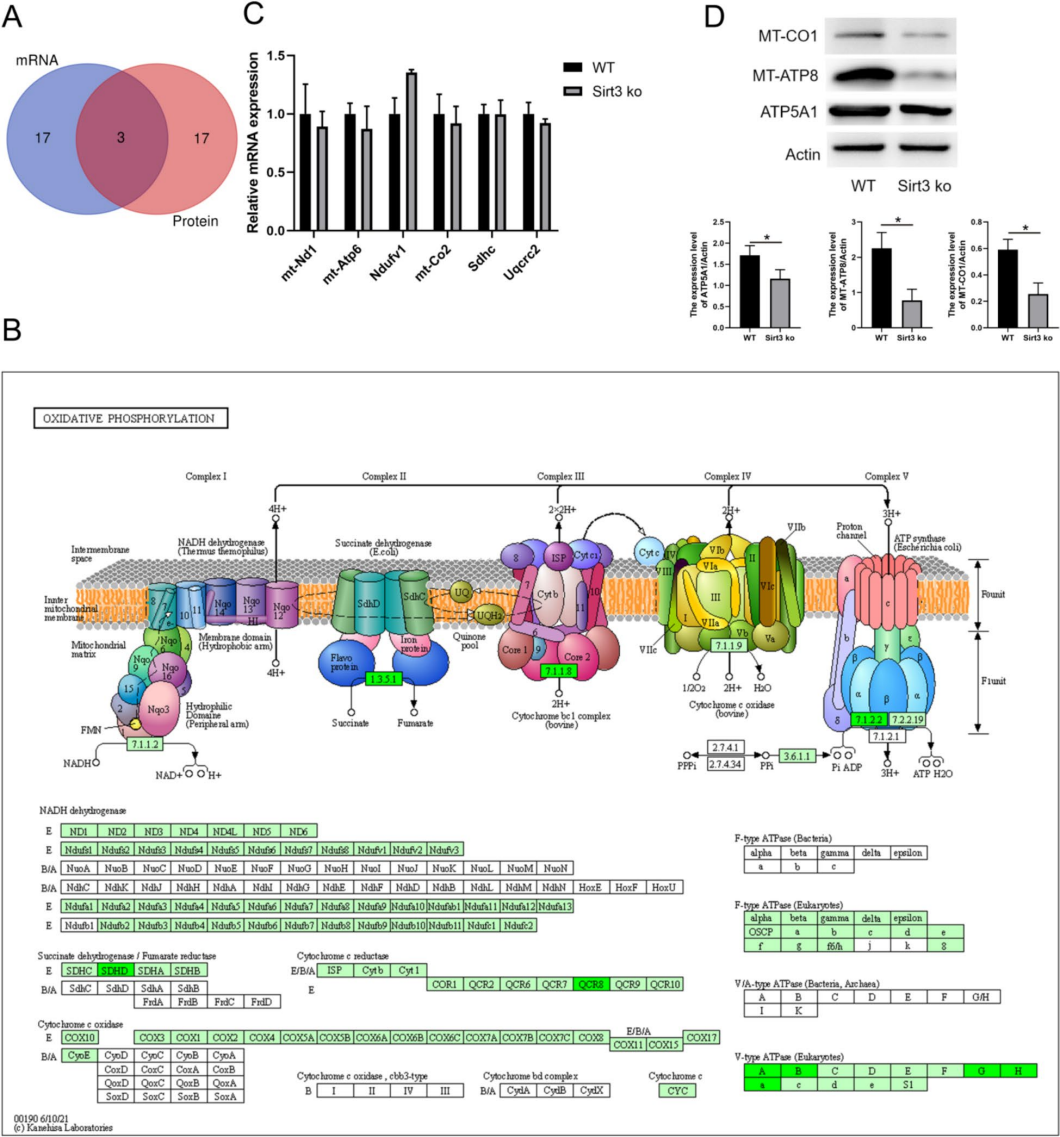
Integrative transcriptomic and proteomic findings suggest that a Sirt3 deficiency inhibits the translation of mitochondrial oxidative phosphorylation complex subunits. **A** Venn diagram illustrates the overlap of the top 20 enriched signaling pathways between transcriptomic and proteomic analyses. **B** KEGG pathway analysis showing protein expression associated with the oxidative phosphorylation complex subunits. **C** Myocardial mt-Nd1, mt-Atp6, Ndufv1, mt-Co2, Sdhc, and Uqcrc2 expression in wild-type and Sirt3-deficient mice as detected by qPCR. **D** Myocardial MT-CO1, MT-ATP8, and ATP5A1 expression in wild-type and Sirt3-deficient mice as detected by western blot and quantified by Image J. Statistical significance: *p < 0.05

Finally, the top 20 pathways from the transcriptomic and proteomic analyses were compared via Venn diagram (Fig. 5A). The oxidative phosphorylation pathway was only enriched in the proteomic data set, with almost all oxidative phosphorylation subunits downregulated (Fig. 5B). To further examine these end points, the mRNA levels of mt-Nd1, mt-Atp6, Ndufv1, mt-Co2, Sdhc, and Uqcrc2 were evaluated by qPCR, showing that all of these subunits of OXHPOS were unchanged (Fig. 5B). To further determine the protein expression of OXPHOS subunits, MT-CO1, MT-ATP8, and ATP5A1 were evaluated via western blot analysis and showed that both of these components were decreased in the Sirt3-deficient mice heart tissues (Fig. 5C). These findings suggest that Sirt3 deficiency can induce oxidative stress by inhibiting the translation of mitochondrial oxidative phosphorylation complex subunits.

### Mitochondrial oxidative phosphorylation dysfunction caused by Sirt3 deficiency verified by metabolomics

**Fig. 6:**
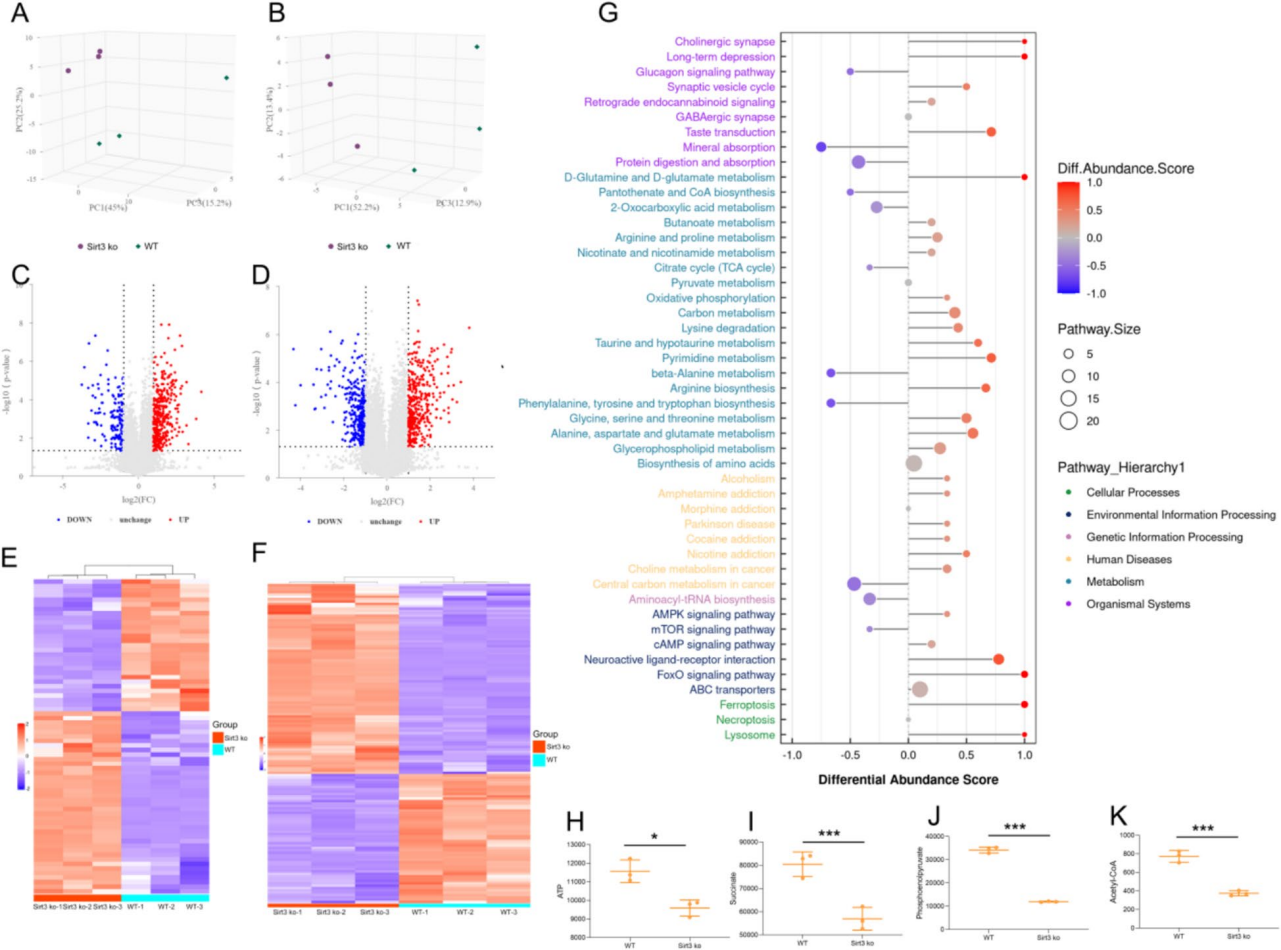
Metabolomic profiles for Sirt3-deficient and wild-type heart tissues. **A** Negative and (**B**) positive ion principal component analysis (PCA) for Sirt3-deficient and wild-type samples. **C** negative and (**D**) positive ion volcano plots displaying differential metabolites between the two groups. Heatmaps showing the expression patterns between the two groups following ESI (**E**) negative and (**F**) positive modes. **G** KEGG pathway analysis comparing metabolic changes between the two groups, with the differential abundance score capturing the average change for all metabolites within a pathway. Statistical graph displaying a subset of differential metabolites that are associated with oxidative phosphorylation, including ATP (**H**), succinate (**I**), phosphoenolpyruvate (**J**), and acetyl-CoA (**I**). Statistical significance: *p < 0.05, ***p < 0.001

To verify that the mitochondrial oxidative phosphorylation dysfunction associated with hypertrophy is caused by a Sirt3 deficiency, metabolomics was employed. Metabolites from Sirt3-deficient and wild-type heart tissues were examined via ESI in positive and negative ionization modes. Both the negative and positive principal component analysis (PCA) results showed that the samples within a group are repeatable, and that the two groups are distinct (Fig. 6A, B). In the negative ion mode, 364 downregulated and 764 upregulated differential metabolites were identified, while in the positive ion mode, 800 were downregulated and 782 were upregulated (Fig. 6C, D). To further examine these profile difference, a heatmap was also constructed (Fig. 6E, F). The KEGG pathway analysis results showed that the differential metabolites were enriched in oxidative phosphorylation, citrate cycle (TCA), cAMP, and mTOR signaling pathways (Fig. 6G). Of the metabolites associated with TCA and oxidative phosphorylation, ATP, succinate, phosphoenolpyruvate, and acetyl-CoA were significantly decreased (Fig. 6H, I). These findings suggest that a Sirt3 deficiency contributes to a disruption in mitochondrial energy metabolism and further confirms that Sirt3 plays a key role in regulating mitochondrial oxidative phosphorylation dysfunction.

### Multi-omics analysis reveals CD38 as the main NAD consumer in Sirt3-deficient mice

**Fig. 7:**
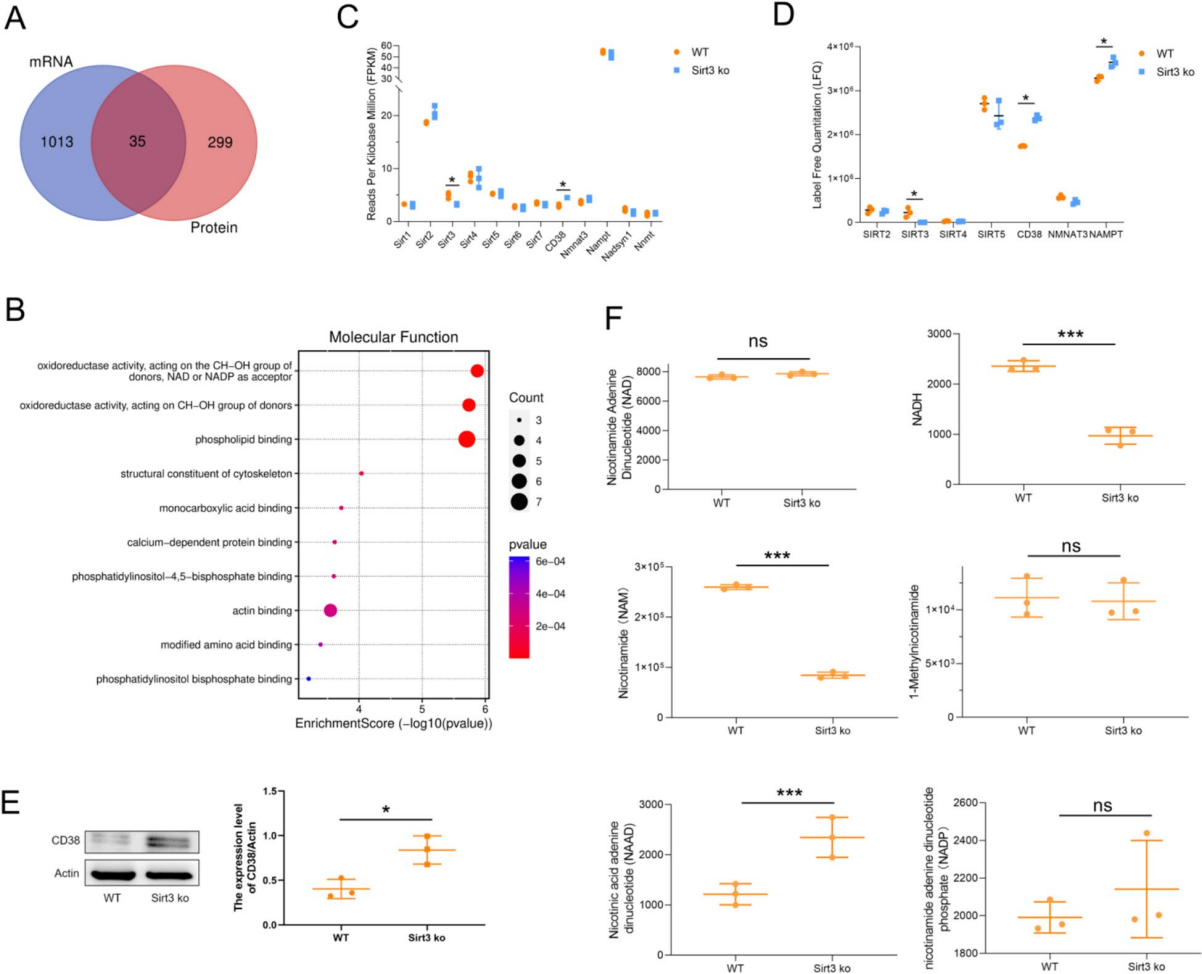
Multi-omics analysis reveals increased NAD consumption and production in the heart tissue of Sirt3-deficient mice. **A** Venn diagram comparing DEGs and DEPs. **B** Molecular functions enrichment analysis of the intersecting genes (*n* = 35). Examination of endpoints associated with NAD synthetases and NAD-consuming enzymes based on the identified DEGs (**C**) and DEPs (**D**). **E** Western blot detects the expression of CD38 in Sirt3 deficient mice heart. **F** Plots examining different metabolites that participate in NAD synthesis and consumption, including NAD, NADH, NAM, NAAD, 1-methylnicotinamide, NAAD, and NADP. Statistical significance: *p < 0.05, ***p < 0.001

Nicotinamide Adenine Dinucleotide (NAD) is a key metabolite in regulating energy metabolism and redox homeostasis, and its content is controlled by NAD synthesis and consumption. While Sirt3 deficiency induces mitochondrial dysfunction, it remains unclear whether or not NAD content is affected. To investigate the effect of Sirt3 deficiency on NAD content, the DEGs and DEPs identified above were compared by constructing a Venn diagram. The results identified 35 overlapping genes (Fig. 7A), with genes found to be associated with the functional group oxidoreductase activity, acting on the CH–OH group of donors, NAD or NADP as acceptor (Fig. 7B). Next, from the 35 overlapping genes, NAD synthetases and NAD-consuming enzymes were screened. The results showed that only CD38 was differentially upregulated at both the transcriptional and translational levels (Fig. 7C, D). We also analyzed the expression of the other NAD synthetases and NAD-consuming enzymes, and the results showed that only Nampt was significantly upregulated at the translational level and the others showed no significant changes (Fig. 7C, D). These results indicate that CD38 is the key regulator of NAD content in Sirt3 ko–induced cardiac hypertrophy.

To further examine the role of NAD in hypertrophy, we screened the metabolites from the metabolomics data set. The results showed that NAD levels were unaltered when comparing Sirt3-deficient mice to wild-type mice (Fig. 7F). However, the content of nicotinamide adenine dinucleotid–hydrogen (NADH) was significantly decreased. In theory, Sirt3 deficiency should reduce NAD consumption, thereby increasing intracellular NAD levels. However, we found that the level of NAD in the myocardial tissue of Sirt3-ko mice did not change. Therefore, we speculate that the synthesis and decomposing of NAD in Sirt3-ko mice cardiomyocytes have been readjusted. To determine the changes in NAD synthesis and decomposition, we analyzed the changes in precursor compounds of NAD synthesis (nicotinamide, [NAM], and nicotinic acid adenine dinucleotide [NAAD]). The results showed that NAM content was significantly decreased, whereas NAAD content was significantly increased (Fig. 7G, H). NAM is a precursor for both NAD and 1-methylnicotinamide synthesis [21]. To determine which product was affected by the decline in NAM, 1-methylnicotinamide levels were also examined. The results showed that 1-methylnicotinamide levels were unaltered in the Sirt3-deficient mice when compared with wild-type mice (Fig. 7I). However, the expression of Nampt, a key catalytic enzyme for NAD synthesis from NAM, was significantly increased. These findings suggest that the decline in NAM is being used for NAD synthesis in the Sirt3-ko samples. Sirt3 deficiency elevates NAD biosynthetic flux, but the steady-state NAD levels remain unaltered, suggesting a simultaneous enhancement of NAD catabolism. Given that CD38 expression was shown to increase in Sirt3-deficient mice and it is an NAD consumer, it would appear that NAD consumption and synthesis are both increased in hypertrophy.

### Sirt3–CD38 axis induces mitochondrial dysfunction through promoting mitochondrial calcium overload

**Fig. 8:**
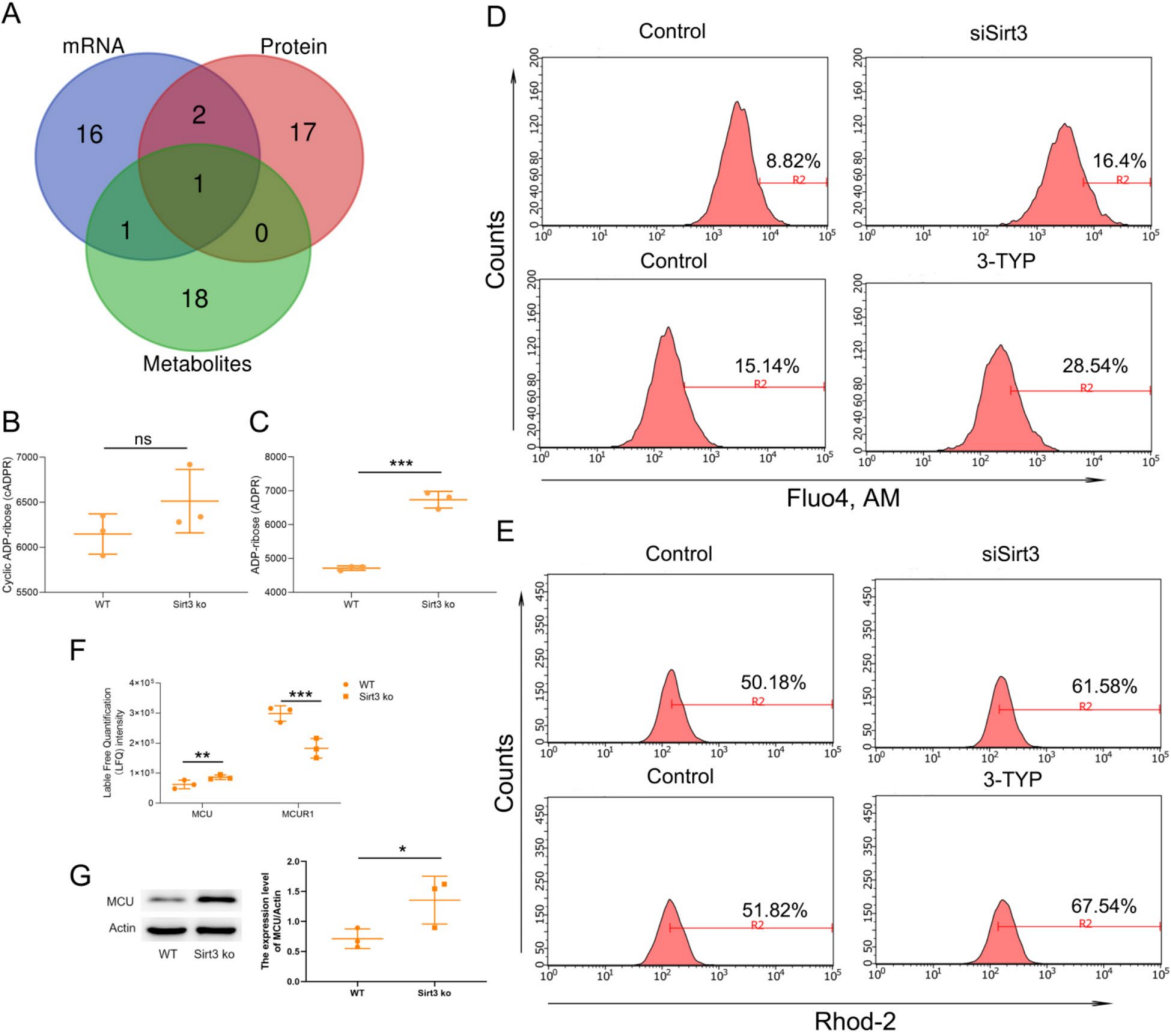
Multi-omics analysis reveals that Sirt3 deficiency induces mitochondrial calcium overload. **A** Venn diagram comparing the top 20 signaling pathways of transcriptomics, proteomics, and metabolomics. **B** and (**C**) plots examining the content of cADPR and ADPR. H9C2 cells were treated with 50 μM Sirt3 inhibitor (3-TYP) for 24 h or transfected with Sirt3 siRNA for 24 h, and then Fluo4, AM (**D**) and Rhod 2 (**E**) was used to label the whole cell calcium and mitochondrial calcium separately. Flow cytometry was used to detect the calcium concentration of cells and mitochondria. **F** Examination of proteins associated with mitochondrial calcium transport (MCU, and MCUR1) in Sirt3 deficient heart. **G** Expression of MCU in Sirt3 deficient heart was validated by western blot. Statistical significance: *p < 0.05, **p < 0.01, ***p < 0.001

To further elucidate the key signaling pathways involved in Sirt3 deficiency-induced myocardial hypertrophy, we conducted Venn analysis on the metabolic pathways of the top 20 of transcriptomics, proteomics, and metabolomics. The results showed that only the cAMP signaling pathway changed simultaneously at all three levels (Fig. 8A). The cAMP pathway, a key regulator of calcium ion levels in cardiomyocytes, plays an indispensable role in regulating the contraction process of cardiomyocytes. In the previous study, we found high expression of CD38, which decomposes NAD to produce NAM and cADPR. cADPR is a key molecule that regulates intracellular Ca^2+^ levels. CD38 can decompose cADPR to produce ADPR and also participates in the regulation of intracellular Ca^2+^ levels. Therefore, we further analyzed the changes in cADPR and ADPR levels through metabolomics data, and the results demonstrated that compared with the wild-type mice, the Sirt3-ko mice did not show any changes in cADPR levels, while ADPR levels were significantly increased (Fig. 8B, C). This further indicates that Sirt3 knockout not only induces CD38 expression, but also the expression of CD38 has high catalytic activity. Appropriate influx of Ca^2+^ into mitochondria is essential for the contraction of myocardial cells, while excessive Ca^2+^ can damage mitochondrial function and induce oxidative stress damage. To determine the changes in intracellular and mitochondrial Ca^2+^ levels, we labeled intracellular Ca^2+^ with Fluo4, and mitochondrial Ca^2+^ with Rhod-2. The changes in Ca^2+^ levels were detected by flow cytometry, and the results showed that after knocking down Sirt3 or inhibiting Sirt3 activity, both intracellular and mitochondrial Ca^2+^ levels significantly increased (Fig. 8D, E). We also analyzed mitochondrial Ca^2+^ transport proteins and found that the expression of mitochondrial Ca^2+^ transport protein MCU was significantly increased, while the expression of inhibitory protein MCUR1 was significantly decreased (Fig. 8F, G).This suggests that Sirt3 deficiency may promote mitochondrial calcium transport through a CD38-dependent mechanism, leading to mitochondrial calcium overload.

**Fig. 9:**
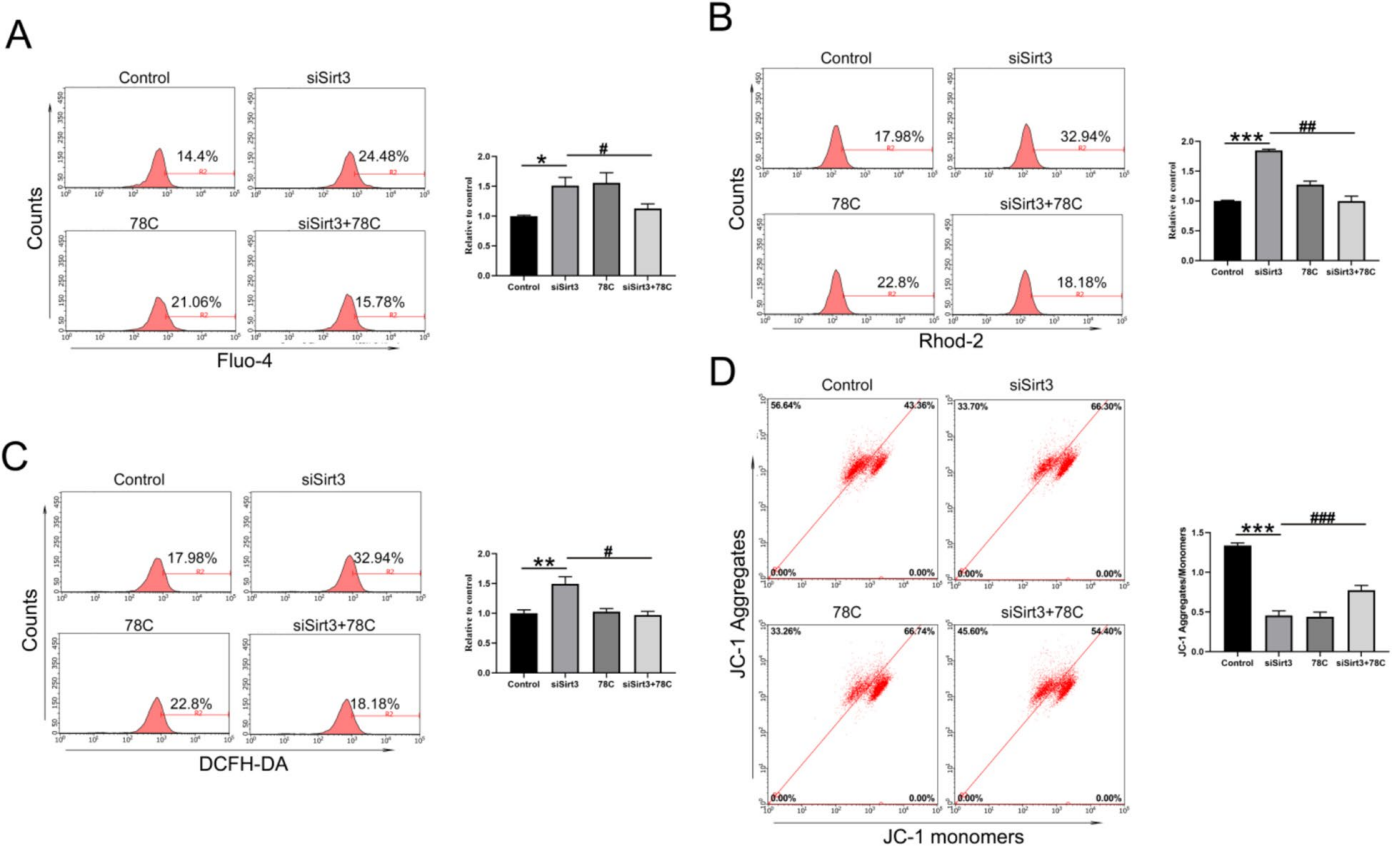
Sirt3–CD38 axis in induces mitochondrial dysfunction by promoting mitochondrial calcium overload. H9C2 cells were transfected with Sirt3 siRNA for 24 h, and then treated with 5 nM CD38 inhibitor (Compound 78 C, 78 C) for 24 h. The whole cell calcium and mitochondrial calcium were labeled with Fluo4 (**A**) and Rhod 2 (**B**) separately, and the calcium concentration of cells and mitochondria were detected by flow cytometry. H9C2 cells were labeled with (**C**) DCFH–DA to determine total ROS levels, or (**D**) JC-1 to assess the MMP via flow cytometry. Statistical significance: *p < 0.05, **p < 0.01, ***p < 0.001 compared with control group; #*p* < 0.05, ##*p* < 0.01, ###*p* < 0.001 compared with siSirt3 group

To further determine whether Sirt3 deficiency induces mitochondrial calcium overload and dysfunction through CD38, we employed siRNA-mediated Sirt3 knockdown combined with CD38 inhibitor treatment in H9C2 cells. The results showed that CD38 inhibition significantly attenuated both intracellular and mitochondrial Ca^2+^ elevation triggered by Sirt3 knockdown (Fig. 9A, B), confirming that Sirt3 deficiency induces mitochondrial Ca^2+^ overload via CD38. In addition, the CD38 inhibitor suppressed Sirt3 knockdown-induced ROS overproduction (Fig. 9C), and restored mitochondrial membrane potential impaired by Sirt3 knockdown (Fig. 9D), indicating improved mitochondrial function. These indicates that the Sirt3–CD38 axis induces mitochondrial dysfunction through promoting mitochondrial calcium overload, and ultimately drives myocardial hypertrophy.

## Discussion

Cardiac hypertrophy refers to pathological remodeling of the heart that occurs when heart cells respond to pathological and physiological stimuli [28, 29]. In the early stages of myocardial hypertrophy, the increase in myocardial mass and strength initially has a beneficial effect aimed at normalizing wall stress and maintaining cardiac output during stress. However, under long-term chronic stimulation, cardiac hypertrophy gradually develops into heart failure. At present, aging is considered one of the most critical independent risk factors for myocardial hypertrophy [30].

Recent studies have shown that all members of the sirtuin family are involved in the regulation of pathological myocardial hypertrophy, and that all except for Sirt4 serve a cardioprotective role [31–35]. The sirtuin family is a class of genes that is conserved across all domains of life from yeast to mammals [12]. In mammals, the sirtuin family mainly consists of seven members that are located in different subcellular organelles [12, 36]. To evaluate the functions of sirtuin family genes, genetic knockout mice were constructed and showed that the absence of sirtuins can result in developmental defects or a shorten lifespan [14, 37]. Moreover, it has been shown that Sirt3 expression is significantly decreased in aging mice, and that knocking out Sirt3 can significantly reduce lifespan in mice [14]. Herein, Sirt3-deficient mice hearts exhibited age-related physiological changes, to include significant increased cardiomyocyte size. In addition, myocardial hypertrophy marker genes, ANP and BNP, were also significantly increased. These findings confirm previous findings that showed that Sirt3 ko mice exhibit pathological myocardial hypertrophy [18].

The heart is one of the organs with the richest mitochondrial content [38]. The abundant mitochondria not only provide the necessary energy for myocardial cell contraction, but also play a crucial regulatory role in maintaining the redox homeostasis of myocardial cells [38, 39]. In cardiac hypertrophy, mitochondrial dysfunction is a major pathological feature that induces energetic impairment and oxidative stress [39]. Herein, Sirt3-deficient cardiomyocytes exhibited a noted decline in mitochondria numbers, a reduction in cristae, an altered matrix and a decreased mitochondrial membrane potential. This mitochondrial dysfunction was also found to be associated with an increase in apoptosis and ROS levels.

Oxidative stress, an imbalance in ROS production and consumption, is considered a key factor leading to cardiac hypertrophy and heart failure. In studies related to the myocardium, it has been shown that oxidative stress activates ROS-sensitive hypertrophy and remodeling signaling cascades [40]. Mitochondria are the main source of ROS, a major byproduct of oxidative phosphorylation, during aging [41]. ROS are cleared by antioxidant enzymes, such as SOD2, catalase, and glutathione peroxidase (Gpx) [40–42]. In a study examining aging-related myocardial hypertrophy in felines, mitochondrial oxidative phosphorylation functioning was impaired and mitochondrial oxidative stress was significantly increased [43].

It has been suggested that Sirt3 not only regulates mitochondrial redox homeostasis, but also participates in regulating the activity of the mitochondrial oxidative phosphorylation complexes, which is crucial for ROS regulation [40, 41]. Moreover, recent studies have shown that activating Sirt3 can inhibit oxidative stress to potential reverse myocardial hypertrophy [37]. In the present study, Sirt3-deficient cardiomyocytes displayed structurally damaged mitochondria, which negatively affected oxidative phosphorylation and increased ROS production. Furthermore, the oxidative phosphorylation dysfunction driven by Sirt3 ko was found to impact oxidative phosphorylation complex subunits and occur at the translational level rather than at the transcriptional level. These findings suggest that Sirt3-induced myocardial hypertrophy during aging may be alleviated by modulating the translation of mitochondrial oxidative phosphorylation complex subunits, thereby mitigating energy metabolism and ROS production. However, the mechanism by which Sirt3 affects the translation of mitochondrial oxidative phosphorylation complex subunits will require further examination and be the focus of our subsequent research.

In a previous study, mitochondrial dysfunction in human hypertrophic cardiomyopathy was associated with myocardial cell structural damage, and this was corrected by improving NADH-driven mitochondrial respiratory inhibition and reducing oxidative stress [44]. As a key deacetylase in mitochondria, Sirt3 reduces intracellular ROS levels by regulating SOD2 activity [12]. Sirt3 is an NAD-dependent deacetylase, and previous studies have found a close correlation between age-related myocardial hypertrophy and decreased NAD levels [21, 45, 46]. NAD is a key metabolite in oxidative phosphorylation and in the TCA cycle, with NAD playing a crucial role in maintaining cellular energy metabolism and redox homeostasis [47]. Furthermore, supplementing with NAD or NAD precursors has been shown to alleviate angiotensin II-induced myocardial hypertrophy [48]. However, in this study, NAD levels were not significantly altered in the Sirt3-deficient samples relative to the wild type, but one NAD precursor, NAM, was significantly reduced, while another, NAAD, was significantly upregulated. Considering that Sirt3 is an NAD-consuming enzyme, an increase in NAD levels was expected in the Sirt3-deficient samples, but yet levels were unaltered. This finding suggested that the Sirt3 deficiency might have activated another NAD-consuming enzyme. After a full multi-omic review, CD38 was isolated as the only elevated NAD-consuming enzyme. Previous studies have shown that CD38 is highly expressed in aging-related myocardial hypertrophy tissues, and that knocking out CD38 can significantly reverse myocardial hypertrophy [21]. The opposite functions between Sirt3 and CD38 in the pathology of cardiac hypertrophy may be caused by the different metabolites during their degradation of NAD. CD38 degrades NAD to produce cADPR, which is a key molecule to mobilize Ca^2+^, and further regulates the cAMP signaling pathway via Ca2^+^ [21]. Our metabolic analysis showed that cADPR was significantly upregulated in the hearts of Sirt3-deficient mice. This may in turn affect calcium ion concentrations in cardiomyocytes and mitochondria, thereby affecting the contractile function of cardiomyocytes [49, 50]. In this study, we found that the PI3K–Akt signaling pathway was significantly changed at the protein level. The PI3K–Akt signaling pathway as the major pathway to regulate translation may be the main reason why mitochondrial OXPHOS complex subunits translation is inhibited by Sirt3 deficiency. Sirt3 degrades NAD to produce O-acetyl-ADP-ribose [25]. Recent studies have suggested that OAADPr may regulate gene silencing by facilitating the assembly and loading of the Sir2–4 silencing complex onto nucleosomes [25, 51]. Sirt3 deficiency results in the decline of OAADPr, which further activates the previously silent genes. Thus, CD38 may be the target of OAADPr, which is activated by Sirt3 deficiency. However, the role of metabolites derived from NAD-consuming enzymes in regulating mitochondrial calcium overload necessitates further in-depth investigation.

In myocardial cells, Ca^2+^ not only regulates excitation–contraction coupling, but also regulates mitochondrial metabolism and oxidative stress signaling, thereby controlling cell function and fate. Indeed, increased cytosolic Ca^2+^ levels with elevated cardiac contractions allow Ca^2+^ to enter the mitochondria and stimulate pyruvate dehydrogenase, α-ketoglutarate, and isocitrate dehydrogenase to regenerate reducing equivalents, as well as signal ATP synthase and complex III of the ETC to accelerate ATP production [1, 3, 4]. Notably, excessive or insufficient mitochondrial Ca^2+^ leads to mitochondrial dysfunction and cardiac pathology [5]. This study showed that Sirt3 deficiency led to an increase in Ca^2+^ concentration in H9C2 cells, and mitochondrial Ca^2+^ concentration in H9C2 cells also increased proportionally. CD38 inhibitor significantly attenuated both intracellular and mitochondrial Ca^2+^ elevation triggered by Sirt3 knockdown, suppressed Sirt3 knockdown-induced ROS overproduction, and restored mitochondrial membrane potential impaired by Sirt3 knockdown. This indicates that Sirt3 deficiency induces calcium overload, leading to mitochondrial dysfunction and increased ROS production, exacerbating myocardial injury. However, there is currently no evidence to suggest that Sirt3 can directly regulate mitochondrial calcium ion levels. Based on our multi-omics analysis results, Sirt3 deficiency affects intracellular and mitochondrial Ca^2+^ concentration by inducing CD38 expression.

Herein, Sirt3-deficient mice were utilized as an age-related myocardial hypertrophy model. Our multi-omics analysis revealed that Sirt3–CD38 axis induced mitochondrial dysfunction through mitochondrial Ca^2+^ overload, which was the core mechanism regulating age-related myocardial hypertrophy. These findings will aid in improving our understanding of the mechanism driving the physiological changes associated with aging-dependent cardiac hypertrophy and provide a potential target for therapeutic intervention.

## Data Availability

No datasets were generated or analysed during the current study.
